# Experimental Modelling of Water-Wave Interactions with a Flexible Beam

**DOI:** 10.12688/openreseurope.17831.1

**Published:** 2024-10-18

**Authors:** Wajiha Rehman, Tim Bunnik, Onno Bokhove, Mark Kelmanson

**Affiliations:** 1 School of Maths, University of Leeds, Leeds, England, UK; 2 Research and Development, Maritime research Institute Netherlands, Wageningen, Gelderland, The Netherlands

**Keywords:** Experimental fluid dynamics, fluid-structure interactions, dynamic response of the flexible structure, maritime engineering.

## Abstract

A series of fluid-structure-interaction (FSI) experiments is presented for studying water-wave interactions with a flexible beam in a wide range of sea conditions, thereby yielding a repository of FSI test-case data. The aim is to use these experimental data in order to validate FSI solvers commonly employed by the maritime industry in the design of fixed-foundation, offshore wind turbines. The experimental set-up allows simultaneous measurements of beam deflections and their effect on incident and reflected waves. In addition, the study is carried out in a wide range of sea conditions ranging from regular-to-irregular and moderate-to-extreme wave height and steepness. The study of such a wide range of conditions makes the experiments suitable for providing reliable data in the validation of a suite of mathematical and numerical FSI solvers, i.e., linear, nonlinear and high-fidelity. The data from the experiments are made publicly available through open-source data-sharing platforms.

## 1. Introduction

Fixed offshore wind turbines (FOWT) are considered an attractive alternative to onshore wind turbines because offshore wind flow is stronger and steadier than on land. Offshore installation additionally circumvents problems related to land availability, noise and interference with communication signals ^1, 2^. However, building FOWT farms is capital-intensive as the design, fabrication and installation of structures in often harsh ocean conditions are challenging. In addition, FOWTs are prone to higher risks of structural damage because they are larger than onshore wind turbines and have to endure hydrodynamic loading in addition to aerodynamic loading ^3^. It is clearly of great importance to predict such loading accurately, which demands a better understanding of the physics of water-wave interactions with a fixed-bottom flexible monopile. The problem of water-wave interactions with such a beam is a complex multiphysics phenomenon known as a fluid-structure interaction (FSI). In FSI problems, the fluid flow interacts with the flexible structure in a way that deforms the structure and, as a result, the structural deformations change the initial fluid flow. Thus the FSI problem is a coupled, two-way problem of which, due to the complexity of the underlying physics, investigation is challenging in terms of experimentation, mathematical analysis and numerical modelling.

In the maritime industry, mathematical and numerical modelling is gaining significance because experimental scaled-model testing is not always feasible in early design stages due to time and budgetary constraints. Moreover, the experimental modelling of flexible structures at the model scale is not straightforward, motivating researchers to develop mathematical and numerical models for solving FSI problems.

These models generally fall into two categories. First, they range from straightforward linear shallow-water equations and linear modal analysis to intermediate-complexity linear potential-flow solvers coupled to linear elastic structural equations ^4, 5^. Second, there are more sophisticated approaches based on nonlinear potential flow, Navier-Stokes (NS) equations ^6^, and Smoothed Particle Hydrodynamics (SPH) ^7^ coupled with nonlinear hyperelastic structural equations. However, results generated by numerical models require validation using benchmark experimental data. The present research therefore concerns wave-basin experiments of FSI problems; specifically, the dynamic response of a flexible beam exposed to (controllably generated) water waves. The aim of the study is the generation of a high-quality experimental data set to be used in the validation of diverse numerical models for solving FSI problems i.e., linear, nonlinear and high-fidelity.

The experimental set-up includes a vertically mounted flexible cylindrical beam equipped with six accelerometers, distributed evenly along its length, that record its dynamic response. Two probes placed at the free surface of the water close to the beam (forward of and to the side of the beam) measure the free-surface elevation of the incident and reflected water waves. The beam is fixed to a basin carriage that traverses horizontally along the wavetank at different speeds so as to control the frequency with which waves encounter the beam. The upper and lower parts of the beam are respectively in air and submerged in water. This model set-up was prompted by the basin depth (3.6m) which excludes the possibility of modelling a bottom-mounted beam. The FSI physics therefore do not exactly resemble those of a FOWT but have sufficient similarity to provide suitable validation material for FSIs of a FOWT. Hammer tests are performed on the beam in air and water to obtain the dry and wetted modes, natural frequencies and structural and hydrodynamic damping. The novelty of the experimental set-up is that it allows simultaneous measurement of beam deflections and their effect on the incident and reflected waves, rendering feasible a study of the FSI problem in diverse-yet-controllable conditions.

The experiments are divided into three cases, each of which is aimed at studying the dynamic response of the flexible beam to varying wave conditions ranging from regular-to-irregular and moderate-to-extreme wave height and steepness. Experimental Case 1 concerns interactions of regular waves with the flexible beam when the carriage is at rest; studying this case will facilitate the validation of linear FSI solvers in the non-resonant regime, since the non-linear dynamic response of beam is not excited by the incident-wave frequencies. Experimental Case 2 concerns interactions with the flexible beam when the carriage is moving at a constant speed. Moving the carriage changes the frequency of encounter between beam and waves, so that the dynamic response of the beam and its interaction with water waves, particularly at the onset of resonance, can be studied. By changing the steepness of the regular waves, both linear and nonlinear FSI solvers can be validated. In this case, the dynamic response of the beam results from an accumulated hydrodynamic loading that cannot be distinguished, by the current experimental set-up, into its consistuent wave- and current-induced components. Experimental Case 3 concerns steep, irregular-wave interactions with the flexible beam when the carriage is at rest. This is the most complex case and is designed to yield data on structural dynamics due to nonlinear wave-loading processes related to steep and breaking waves. This case will help to validate the high-fidelity FSI solvers.

Hence, the study covers a wide range of FSI problems that can be used to establish benchmarks for FSI-code validations.

## 2. Design of experimental set-up

**Figure 1.**
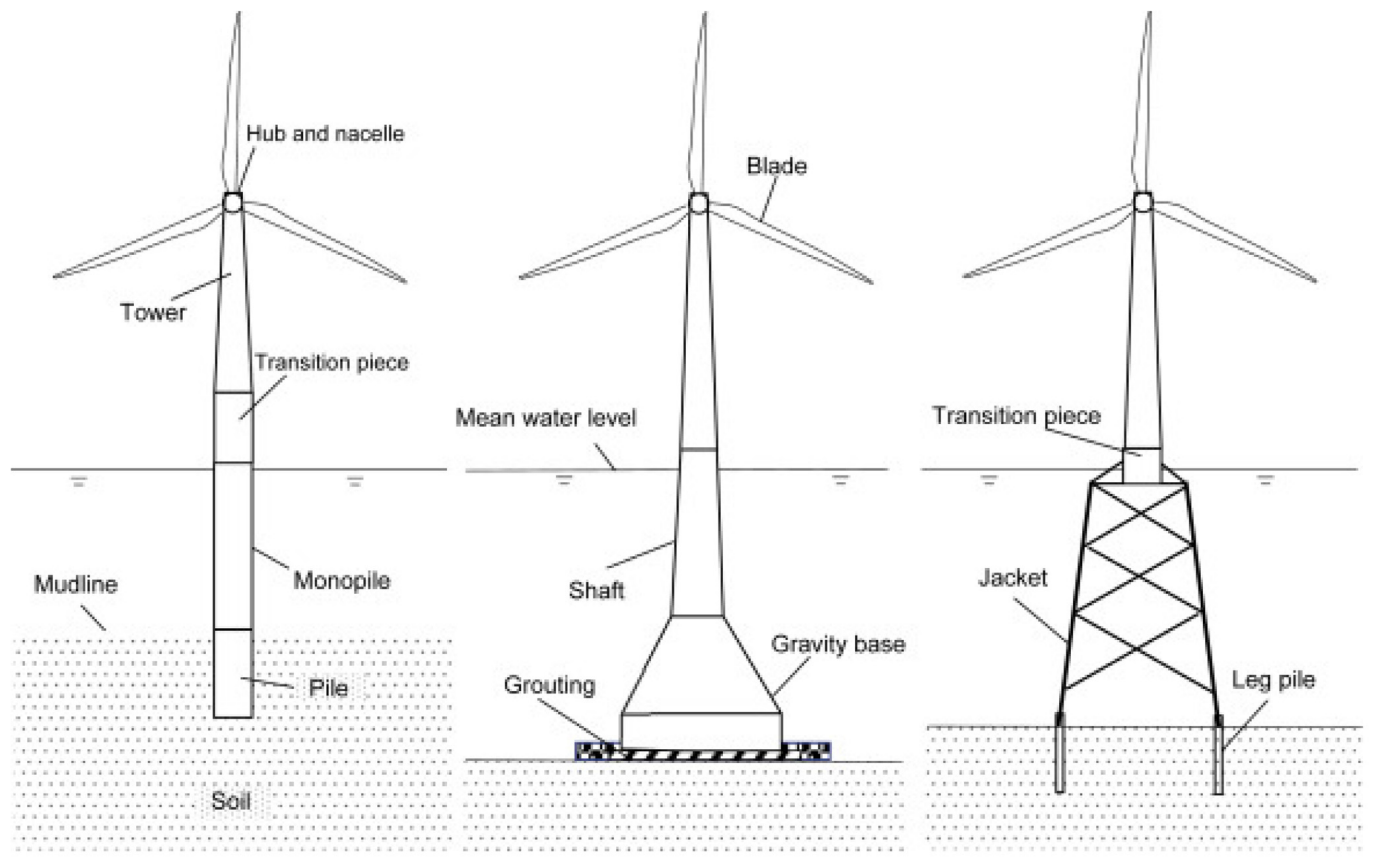
Schematic of different fixed-bottom OWT foundations; monopile, gravity-based and jacket. Copyright © 1969, Elsevier ^9^.

The experimental set-up and laboratory facilities are now explained. The FSI set-up is designed to mimic the (simplified by neglecting the rotor effects) physics of a fixed-bottom offshore wind turbine (OWT) mast; i.e. the focus is solely on the response of the flexible mast to water-wave loading and the concomitant changes in fluid flow due to the mast’s deformations. Such a rotorless set-up will hopefully admit extensions aimed at broadening the application of the experimental data to other FSI problems; for example, in the design of vortex bladeless wind turbines ^8^. Fixed-bottom OWTs occur in three forms, defined by their foundations, as shown in Figure 1.

Of the three types of OWT foundation, the monopile is the subject of the present experimental study as it has the simplest design; one that comprises a single steel-tube pile. Before designing the experimental set-up, it is important to have a basic understanding of the (rotorless) dynamics of the mast of the monopile fixed-bottom OWT. Study of soil-structure interactions ^10^ confirms that the overturning moment generated at the mast’s bottom, due to the wind and water-wave loading acting on the mast, causes angular movement of the buried (in soil) section of the foundation. Therefore, this behaviour should be incorporated into the experimental set-up.

**Figure 2.**
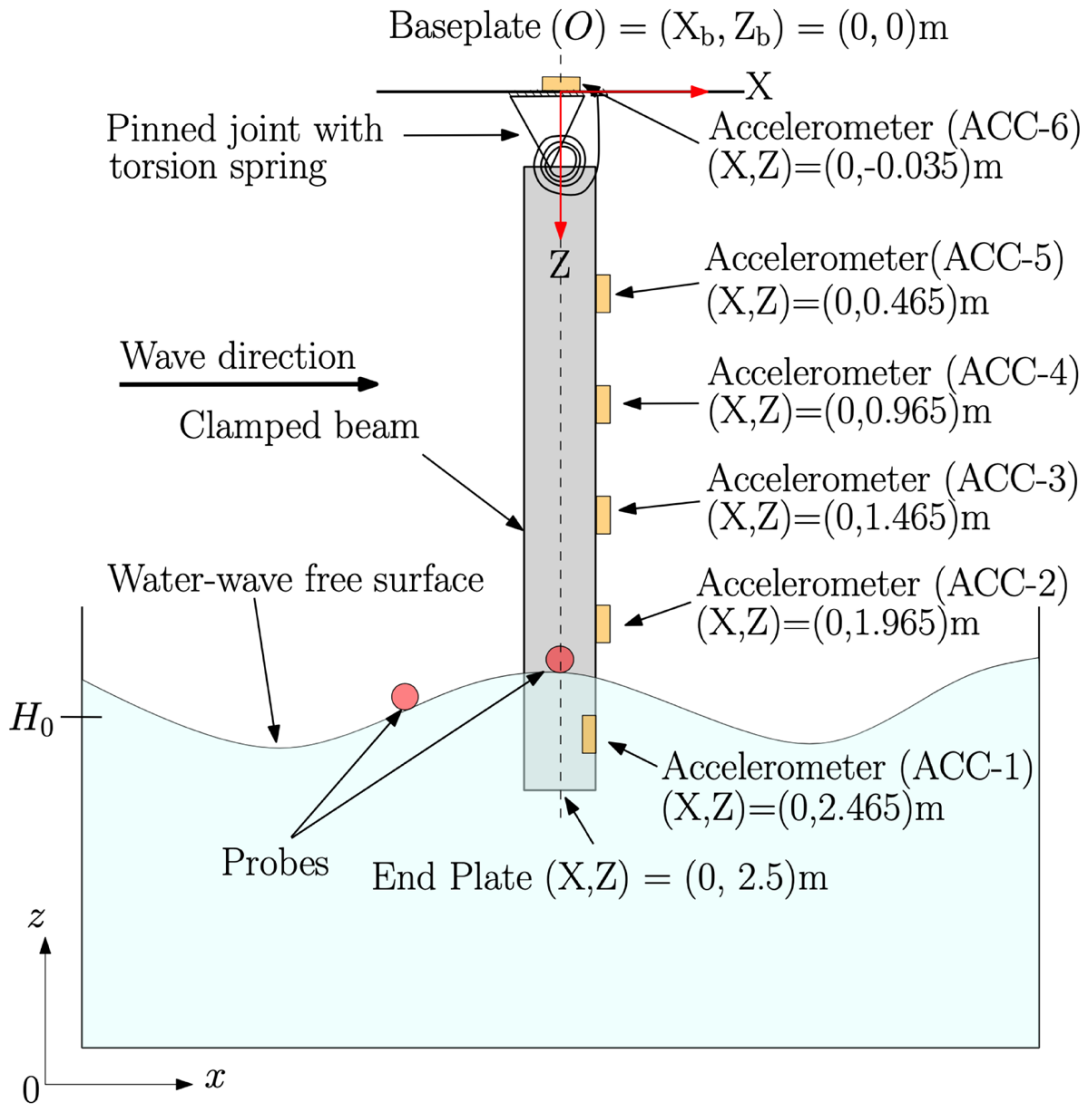
Schematic side view of the experimental set-up. An Eulerian-coordinate system (denoted by *x, y* and *z*) is used for the wavetank; its origin ( *x, y, z*) = (0, 0, 0)m is located in the middle of the wavemaker at rest. A Lagrangian-coordinate system at rest (denoted by X, Y and Z) is used for the beam; its origin is at the base plate (labelled O in the figure) (X, Y, Z) = (0, 0, 0), which origin has fixed Eulerian position ( *x _b_, y _b_, z _b_*) = (30, 2.05, 4.6)m. At rest, the end plate at the free submerged end of the beam is located at (X, Y, Z) =(0, 0, 2.5)m. The base plate is flexible enough to allow rotation of the beam, represented by a pinned joint with a torsion spring. Moreover, the submerged accelerometer is internal. A more detailed CAD drawing of the set-up with exact dimensions and location of the sensors can be found on GitHub.

The experimental set-up, a schematic side view of which is shown in Figure 2, comprises an initially vertical flexible cylindrical beam, one (top) end of which is fixed to a basin carriage having a base made of PVC that is flexible enough to allow angular motion of the top of the beam, yet at the same time strong enough to keep the assembly intact. The other (bottom) free end – initially vertically below the fixed end – is submerged in water. The basin carriage can traverse along the basin’s length at different speeds. There are six equidistant accelerometers attached along the beam’s length for measuring the beam’s acceleration. Five out of six accelerometers are attached to the outer surface of the beam while the sixth one is attached to the inner surface of the submerged end of the beam. This is done to eliminate the interaction of the accelerometer with the water waves. Additionally, two probes (indicated by red discs in Figure 2) are placed at the water free-surface, in the vicinity of the beam, to measure the wave elevation of the incident and reflected waves from the beam; the two probes are located at ( *x*, *y*, *z*) = (26.25, 1.475, 3.6)m and (30, 1.475, 3.6)m from the wavemaker, where *x* is the distance along the length of the wavemaker and *y* shows the distance in the lateral direction from the centre of the wavemaker. This set-up admits simultaneous measurement of beam deflections and their effect on the incident and reflected waves and hence facilitates a quantifiable study of the FSI problem in a controlled environment.

**Figure 3.**
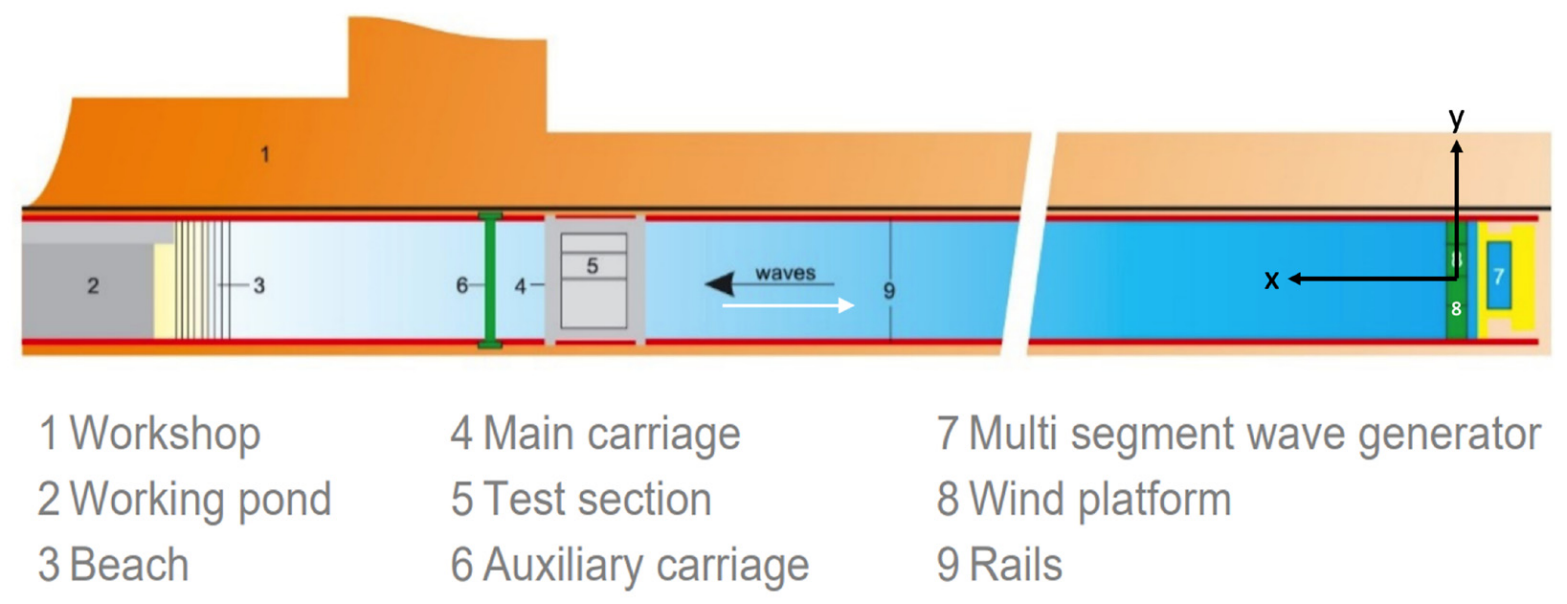
Schematic plan view of the concept wave basin at MARIN, The Netherlands ^11^.

Experiments have been conducted in the concept-design basin at the Maritime Research Institute Netherlands (MARIN). The concept basin is a 220m-long, 4.01m-wide and 3.6m-deep rectilinear basin filled with fresh water. It has a stiff carriage that can traverse along the basin’s length at a maximum speed of 10m */*s. At one end of the basin, there is a flap-type wavemaker that has eight contiguous paddles. The wave generator has the capacity to generate waves up to a significant wave height of 0.55m, at a peak period of 2.3s. A schematic plan view of the basin is shown in Figure 3.

First, parameters for generating a required theoretical waveform are given to the wave-maker and the waves generated experimentally are measured by probes and compared with the required waveform. The difference between the experimental and required waves is used to adjust the wavemaker to obtain the required wave.

However, a difference of up to 5% may still accrue between desired and iterated wave-forms, but this is not an issue since the undisturbed waves ultimately used in the experiments are recorded. The wave parameters, i.e. wavelength Λ and wave height *H*, can be calculated using the dispersion relation for deep-water dynamics i.e., water depth *d >* Λ */*2 (which applies here), given as


$$\;\omega^{2}=gk,\;(1)$$

where *g* is gravitational acceleration, and the wave number is *k* = 2 *π/*Λ, so that wavelength and period are related via


$$\;\text{Λ}=\frac{g}{2\pi }T^{2}.\;(2)$$

Formula (1) and Formula (2) are used to compute wave parameters Λ and *ω*, values of which are given in the following descriptions of experimental cases.

### 2.1 Beam selection and procurement

**Figure 4.**
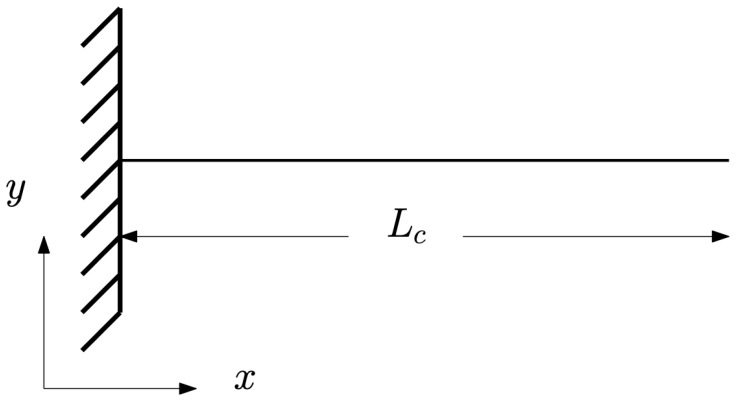
Two-dimensional view, in the *x, y* plane, of a one-dimensional cantilever beam of length *L _c_* ^12^.

The first significant experimental-set-up step is the selection of a beam flexible enough to model the FSI problem yet stiff enough to maintain a straight vertical position in the absence of external loading. After considering different material parameters, costs, and market availability, a cylindrical beam made of polyvinyl chloride (PVC) was selected. Beam dimensions were decided by calculating the natural beam frequency for different values of chosen parameters of length, wall thickness, and diameter. This parametric study is based on analysis of the horizontal cantilever beam shown in Figure 4 and given as the clamped, free-beam case in 12, Table 8-1. Note that the *x, y* coordinates in Figure 4 differ from those used in the FSI experiments, the latter being used solely for referencing the beam geometry. Since the beam in the experimental set-up hangs vertically, any horizontal deflections from rest will be small, and the impact of water waves in the actual experiments will dominate over the restoring force of gravity, which is accordingly ignored in the analysis.

The goal being to select the values of the aforementioned parameters such that the beam’s natural frequency lies outside the short, primary-wave regime. In this way, natural modes of the beam could not be excited by linear-wave effects, thereby admitting the study of the nonlinear response of the beam. By moving the carriage into the waves, the wave-beam-encounter frequency of the waves can be tuned to match the natural frequency of the beam; in this way, the linear resonant response can also be studied. The empirical formula for the natural frequency *f _i_* of the *i* ^th^ mode of the cantilever beam is given by


$$\;f_{i}=\frac{\lambda_{i}^{2}}{2\pi L_{c}^{2}}{\left(\frac{E_{c}I_{c}}{M_{c}}\right)}^{1\text{/}2};\;i=1,\;2,\;3,\;\ldots \;,\;(3)$$

where *L _c_* is the length of the cantilever beam; *E _c_* is the elastic modulus and *M _c_* its mass per unit length of the cantilever beam’s material, and


$$\;I_{c}=\frac{\pi }{4}\left(a_{c}^{4}-b_{c}^{4}\right)\;(4)$$

is the area moment of inertia of the tubular beam with outer and inner radii *a _c_* and *b _c_* respectively. The modal profile *ỹ* corresponding to the *i* ^th^-mode of the cantilever beam is given by


$$\;\begin{array}{l} {\tilde{y}}_{i}=\text{cosh}\frac{\lambda_{i}x}{L_{c}}-\text{cos}\frac{\lambda_{i}x}{L_{c}}-\sigma_{i}\left(\text{sinh}\frac{\lambda_{i}x}{L_{c}}-\text{sin}\frac{\lambda_{i}x}{L_{c}}\right);\\ \;i=1,\;2,\;3,\;\ldots \; \end{array}\;(5)$$

where *x* is the distance from the fixed end, and the eigenvalues λ *_i_* and *σ _i_* of the cantilever beam, corresponding to each mode number *i*, are real numbers calculated by Blevins ^12^ using the modal-analysis method of vibration response: their values for a cantilever beam are shown in Table 1, which is taken from 12.

**Table 1.** Eigenvalues *λ _i_* and *σ _i_* of the cantilever beam, from Table 8-1 of 12.

Mode number ( *i*)	*λ _i_*	*σ _i_*
1	1.87510407	0.73409551
2	4.69409113	1.01846732
3	7.85475744	0.9992245
4	10.9955407	1.00003355
5	14.1371684	0.99999855
*i* > 5	(2 *i* – 1)π/2	≈ 1

The formulas ( 3)–( 5) and parameters (i.e. Ec, Ic, Lc and Mc) are for a cantilever beam set-up that allows us to obtain initial guesses for the natural frequency and dimensional parameters of the beam that would be actually used in the study. Now the beam material has been selected, we continue considering the FSI set-up of Figure 2. A PVC baseplate attached to the beam allows it to be mounted to the wavetank carriage. The baseplate additionally admits cables to be connected to the sensors in such a way that interaction with any beam displacements is minimised as much as possible. The free (submerged) end of the beam is sealed with a PVC circular end plate such that water cannot enter the hollow beam.

**Figure 5.**
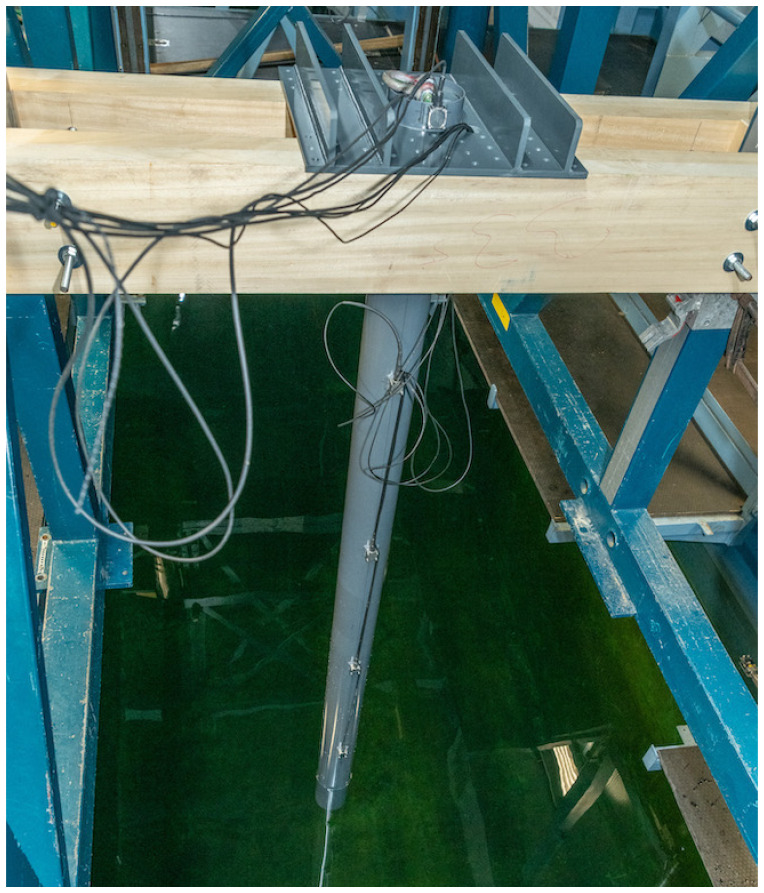
Baseplate, wooden support, beam, accelerometers and cables of the beam. See text for details.

The described set-up is shown in Figure 5. The masses and locations of accelerometers and end plate are given in Table 2. The total mass of the beam with accelerometers and baseplate is 4.66kg. The accelerometers (ACC) are numbered from 1 to 6 where ACC-1 is the accelerometer attached at the submerged free end and ACC-6 is attached at the fixed end of the beam. Calculations using these data give an effective mass per unit length of *M* = 1.3552kg/m. Parameters for the beam chosen for the experiments are given in Table 3. 

**Table 2.** Masses and locations of experimental furniture. The position of baseplate is used as a reference for the distances in the second column.

	Distance from baseplate [m]	Mass [kg]
ACC-6	-0.035	0.079
ACC-5	0.465	0.079
ACC-4	0.965	0.079
ACC-3	1.465	0.079
ACC-2	1.965	0.158
ACC-1	2.465	0.079
End plate	2.5	0.15

Although the purpose of these calculations is to obtain an estimate of the beam’s material and dimensional parameters and dynamic response, the actual parameters and responses are better determined by performing hammer tests, as the calculations do not take into account factors such as the weight of sensors and cables, and unavoidable deviations in material properties accrued during manufacturing and fabrication processes. Hence, material parameters and dynamic responses of the beam assembly are determined by performing hammer tests, as described next.

**Table 3.** Beam parameters in the FSI experiments.

Parameter	Value [mm]
Outer diameter (2 *a*)	125
Inner diameter (2 *b*)	120
Thickness ( *a – b*)	2.5
Length ( *L*)	2500

## 3. Hammer tests on the beam

A hammer test is an experimental method for determining a structure’s response and measuring its frequency response function (FRF). An impulse force is applied to excite the structure at a wide range of frequencies and the response is measured using accelerometers. The purpose of exciting the structure at a wide range of frequencies is to obtain its resonance frequencies. The obtained response can then be analysed in the frequency domain to determine dynamic parameters such as stiffness, mass and damping; modal parameters such as natural frequency and mode shapes; and, material properties of the structure. FRF, also known as the accelerance, is defined as the ratio of the output response (here accelerations) and input (impulse force) ^13^. FRF therefore has dimensions of inverse mass and the units of the input and output signals determine the units of the FRF. For example, if the input signal is in units of force (N) and the output signal is in units of acceleration (m/s ^2^), then the FRF will have units of kg ^−1^. However, in this article, we have computed neither FRF nor accelerance, their mention being only for information.

The response is obtained in terms of time-domain signals, here the sensor accelerations, that can be subsequently integrated to yield either velocity or displacement. The output is measured at different positions along the beam, while the input force is applied at a specific position. Hammer tests are performed to obtain the natural periods of the beam, which are used to calibrate the wave frequency required to excite the beam at that period. Exciting the beam at its natural period results in large deformations of the beam, which can be used to validate FSI solvers against nonlinear (hyperelastic) structural solvers.

Dry hammer tests of the beam assembly shown in Figure 5 are conducted by lifting the beam in the air and applying an impulse force with a hammer, upon which dynamic responses (accelerations) of the beam are measured by the accelerometers. Wet hammer tests (of direct relevance to FSI studies) are performed in order to study the effect of submerged beam length on its response. It is found that the resonance time period of the beam increases with increasing submergence of the free end of the beam since the increasing submergence raises the hydrodynamic damping coefficient thereby effectively adding mass to the beam. This is termed *added mass* and refers to the inertia added to the system due to the fluid volume displaced by a submerged motion. Based on the hammer-test study, two submergence depths, of 0.25m and 0.5m, are used in the experiments since the resonance time periods of the beam for these depths are achievable using the waveflap wavemaker at the facility.

### 3.1 Results from dry and wet hammer tests

**Figure 6.**
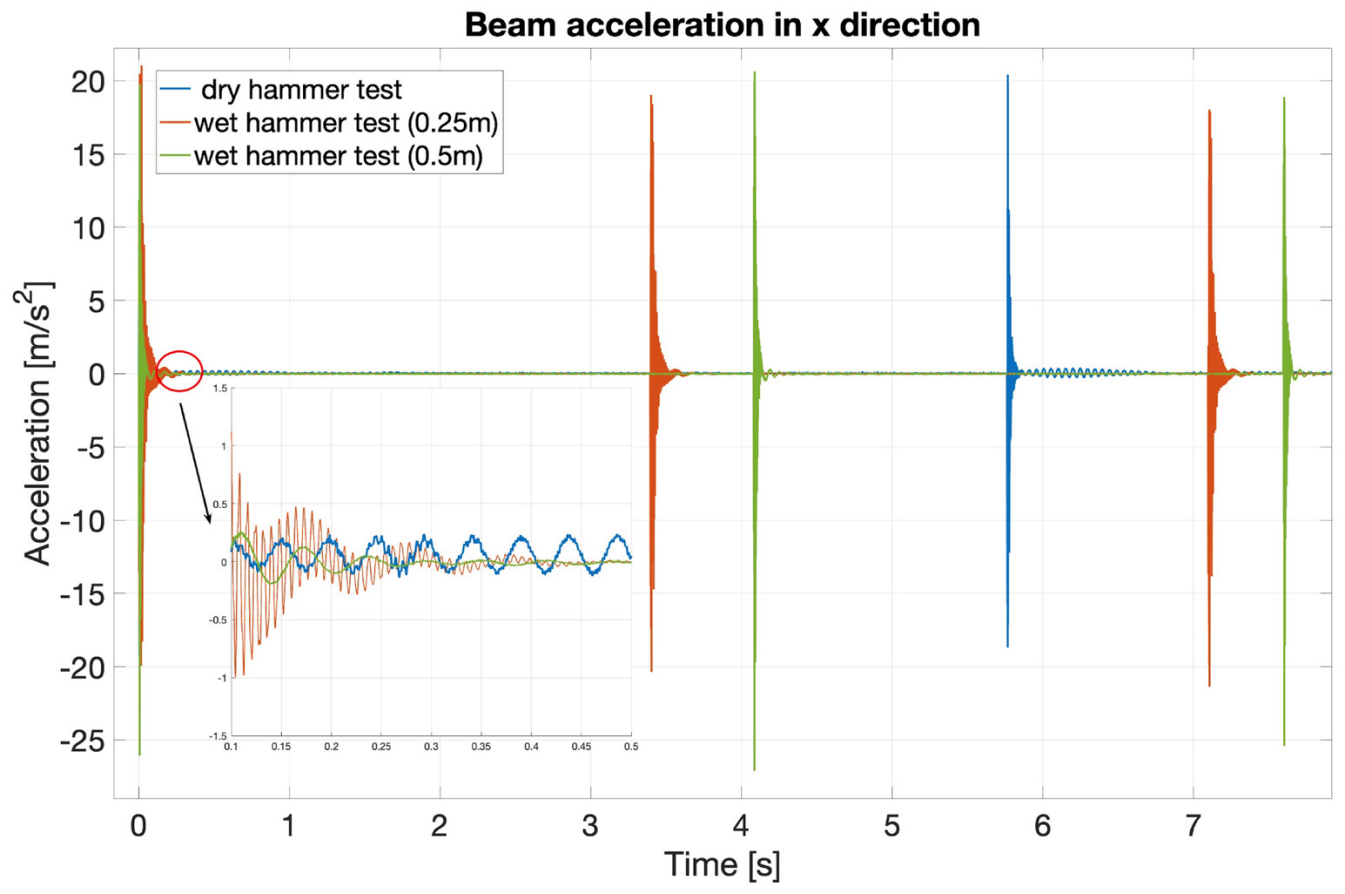
Time-domain beam responses (accelerations in x direction) for the three hammer tests. Dry (blue), wet (red, 0.25m-deep) and wet (green, 0.50m-deep) tests.

Time-domain beam responses obtained from both dry and wet hammer tests (the latter, at two different submergence depths) are presented in Figure 6. Each test comprised three hammer strikes on the beam. Hence, there are 9 peaks in total; three for each test. For the purpose of graphical comparison, the extra signal before the first peak is manually excluded so all peaks can coincide at the start of the signal. A zoomed portion of the comparison is shown in Figure 6. The second blue reading is hidden behind the second red peak. Comparison of the three initial peaks appears in the expanded “early” inset in Figure 6, which reveals that the degree of submergence affects the time-period of the signal.

**Figure 7.**
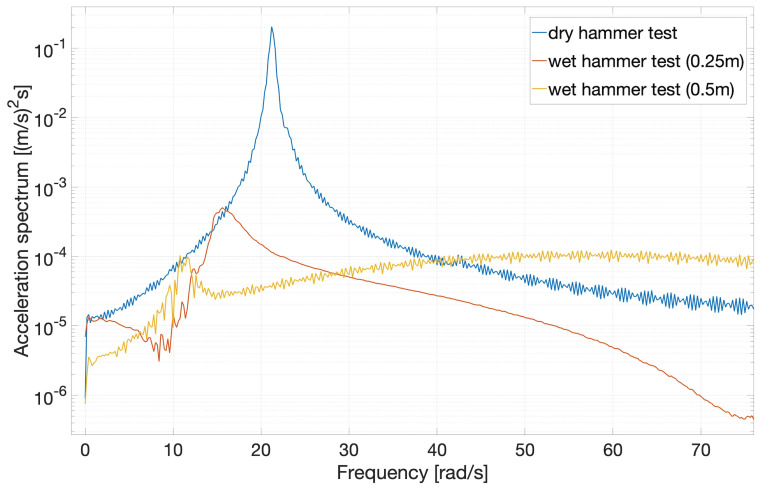
Frequency-domain beam-response spectra for the three hammer tests.

**Figure 8.**
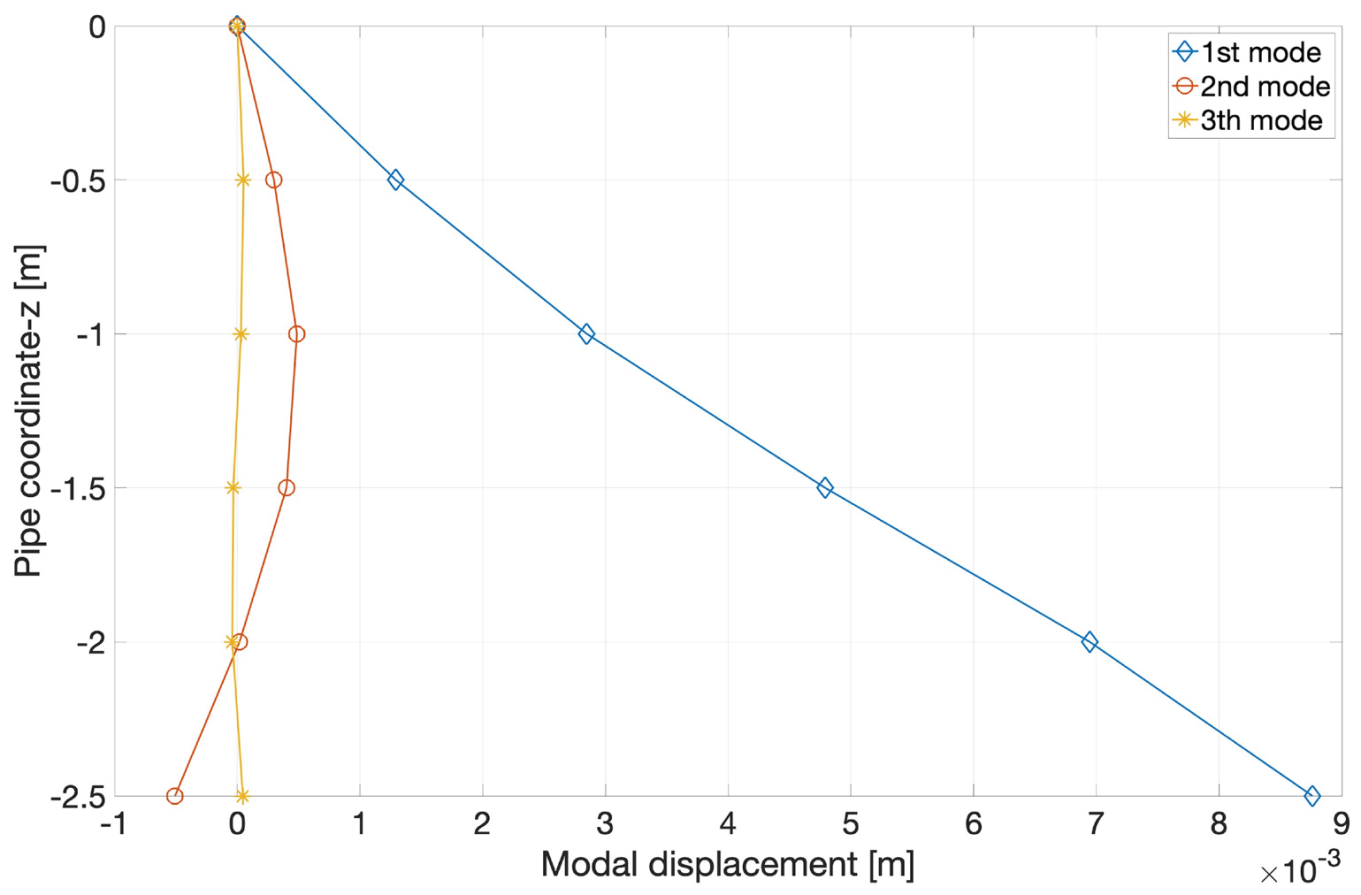
Profiles of first three beam modes, integrated from sensor accelerations measured in dry hammer tests.

Figure 7 shows the frequency-domain dynamic beam response for the three hammer tests. The peaks show the frequencies of the dominant modes of the beam for each test. The reduction in peak frequency in the hammer-test sequence dry (blue), wet (red, 0.25m-deep) and wet (yellow, 0.50m-deep) is clearly consistent with the above-mentioned increase, with submerged depth, of both damping and effective beam mass. In addition, Figure 8 shows the first three modes of the beam calculated by integrating the accelerations obtained in the hammer tests.

Finally, we compute the time periods and natural frequencies of the beam responses, measured in the hammer tests, by converting the time-domain signal into the frequency domain using the MATLAB functions for Fast-Fourier Transform (FFT), Direct Fourier Transform (DFT), and Cross Spectral Density (CSD) methods. Each type of hammer test (one dry and two wet) was performed twice and the values of frequency and time period of the measured accelerations over time are shown in Table 4, each augmented by an error tolerance. Error tolerance is calculated by taking the standard deviation *σ* of the fundamental frequency calculated by using six values, the three ‘FFT’s’ for two repeat tests. 

**Table 4.** Natural frequency and time period of the beam’s first mode, from accelerometer data in hammer tests.

	Period	Natural Frequency	Natural Frequency
	[s]	[s ^−1^]	[rad/s]
Dry hammer test	0.28 *±* 0.002	3.6 ∓ 0.03	22.62 ∓ 0.19
Wet hammer test (0.25m)	0.43 *±* 0.037	2.34 ∓ 0.2	14.70 ∓ 1.26
Wet hammer test (0.5m)	0.58 *±* 0.028	1.72 ∓ 0.09	10.81 ∓ 0.53

The values of the resonant time period and natural frequency measured from the hammer tests confirm that the resonant time period of the beam increases when the beam is submerged in the water due to an increase in added mass and damping coefficient. Table 4 confirms this assertion. Such errors are propagated into subsequent calculations by using the mean, of the implied extreme values, on which a symmetric error range is centred.

**Figure 9.**
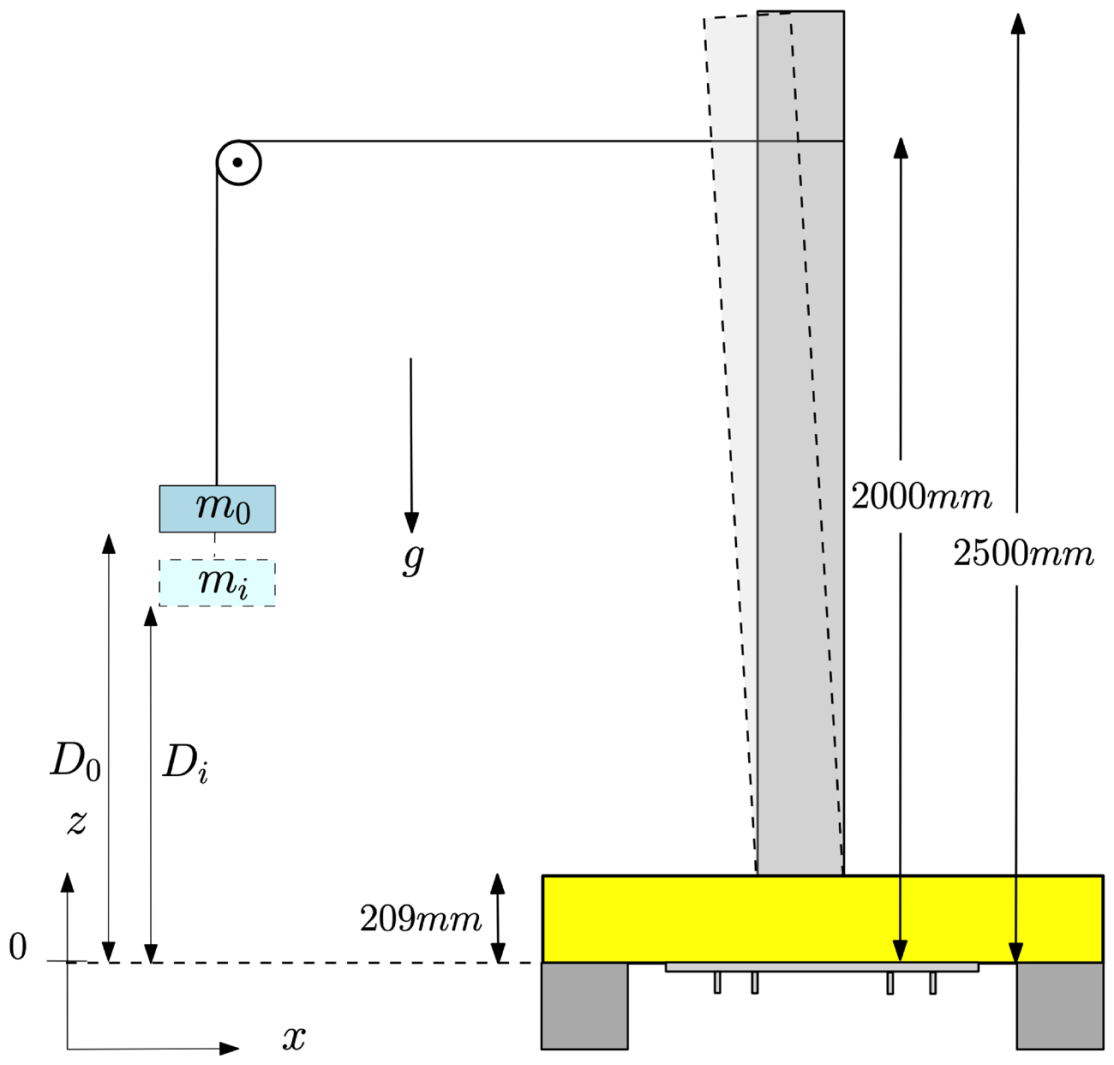
Schematic diagram of the bending test. The beam before deflection is shown as a dark-grey rectangle. The beam deflected by loading of mass *m _i_* appears as a the light-grey curvilinear quadrilateral. Movement of the base is precluded by clamping the base plate with rigid wooden blocks (shown in yellow) in such a way that the beam can move freely in the *x*-direction.

The elastic modulus of the beam is measured experimentally by performing the bending test with the beam while its fixed end is clamped. The bending test consists of applying a gradually increasing known force *F _i_* = *g × m _i_* at a point *L _p_* = 2.0m from the clamped end of the beam and then measuring the beam’s increasing deflection as 1kg masses are sequentially stacked on top of each other on a string attached to the beam’s free end. The schematic of the bending test is shown in Figure 9. 

Each distance *D _i_* in Table 5 is measured from the bottom of the plate at which mass *m _i_* is placed. These masses are in the form of circular iron disks that are stacked on top of each other as described above. The deflection or static offset of the beam is the difference between the two consecutive values of measured distances, i.e.

**Table 5.** Dependence of deflection *ζ _i_* and maximum static offset *δ _i,max_* of beam on increasing mass-loading *m _i_*.

*i*	Mass	Force	Distance	Deflection	Maximum deflection
	*m _i_*	*F _i_*	*D _i_*	*ζ _i_*	*δ _i,max_*
	[kg]	[N]	[mm]	[mm]	[mm]
0	0	0	519	0	0
1	1	9.81	512	7	7
2	2	19.62	506	6	13
3	3	29.43	500	6	19
4	4	39.24	495	5	24
5	5	49.05	488	7	31
6	6	58.86	481	7	38
7	7	68.67	476	5	43
8	8	78.48	470	6	49
9	9	88.29	464	6	55
10	10	98.1	458	6	61
11	11	107.91	452	6	67


$$\;\zeta_{i}=D_{i-1}-D_{i};\;i=0,\;1,\;\ldots \;,\;11.\;(6)$$

The measured distances and deflections corresponding to the applied point loads are listed in Table 5.

The flexural rigidity *EI* of the beam is given by


$$\;EI=\frac{F_{max}L_{p}^{3}}{3\delta_{max}},\;(7)$$

where *F _max_* is the maximum applied point force and *δ _max_* is the corresponding maximum deflection or static offset. Using the experimentally determined natural frequencies (and hence periods) given in Table 4, and the elastic modulus *E* of the material computed using ( 7), the spring constant *k* of the torsional spring shown in Figure 2 can now be calculated, by using the procedure formulated by Blevins ^12^, as follows. The expression for the natural frequency of the pinned free beam with a torsion spring at the pinned joint is given in 12 as


$$\;f_{i}=\frac{\lambda_{i}^{2}}{2\pi L^{2}}{\left(\frac{EI}{M}\right)}^{1\text{/}2};\;i=1,\;2,\;3,\;\ldots \;,\;(8)$$

where *f _i_* is the fundamental frequency of the *i* ^th^ mode (computed via hammer tests), *M* is the mass per unit length (computed via the mass-distribution information in Table 2), *L* is the length (measured), *EI* is flexural rigidity (computed via a bending test), and λ *_i_* is obtained from Table 6, which displays the data given in Blevins ^12^.

**Table 6.** Natural frequencies of a pinned free beam with a torsion spring at a pinned joint. *λ _i_* is a function of *kL/*( *EI*). Table reproduced from 12, in which data are provided to 4 significant figures.

*kL/EI*	*λ _i_* ( *kL/EI*) *i* = 1
0	0
0.01	0.4159
0.1	0.7357
1	1.248
10	1.723
100	1.857
*∞*	1.875

**Figure 10.**
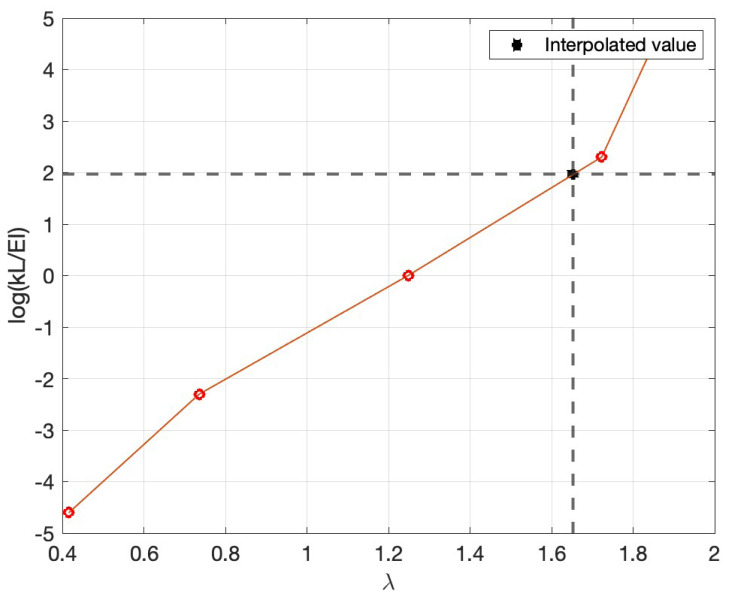
Semilog plot of data in Table 6 on which linear interpolation of *kL/EI* is performed, as described after ( 9) in the text.

For the given material parameters, the stiffness *k* of the moving base, represented by the torsional spring in Figure 2, is derived as follows; first, λ is calculated by rearranging ( 8)


$$\;\lambda =\sqrt{\frac{f2\pi L^{2}M^{1\text{/}2}}{{\left(EI\right)}^{1\text{/}2}}}=1.65\pm 0.01,\;(9)$$

which value of *λ* is then used to find the corresponding value of *kL/EI* from Table 6 via linear interpolation when the ratio *kL/EI* is converted onto a logarithmic scale, as shown in Figure 10. This yields


$$\;\text{log}\left(\frac{kL}{EI}\right)=1.96\pm 0.03\;(10)$$

from which the logarithmically interpolated *kL/EI* is 7.13 *±* 0.24. Finally, the stiffness or torsional spring constant *k* is then computed as


$$\;\begin{array}{c} k=\left(7.13\pm 0.24\right)\frac{EI}{L}\\ \;=\left(12.24\pm 0.41\right)\times 10^{3}\mathrm{Nm/rad}. \end{array}\;(11)$$

All material parameters of the beam are summarised in Table 7.

**Table 7.** Material parameters of the beam used for the FSI experiments. Error tolerances are not available for all parameters.

Parameters	Units	Values
Spring Stiffness ( *k*)	[Nm/rad]	(12.24 *±* 0.41) *×* 10 ^3^
Elastic Modulus ( *E*)	[N/m ^2^]	2.378 *×* 10 ^9^
Mass per length ( *M*)	[kg/m]	1.6048
Length ( *L*)	[mm]	2500
Density	[kg/m ^3^]	1668

## 4. Case-1 experiments: interactions of regular waves with the flexible beam when the carriage is at rest

**Figure 11.**
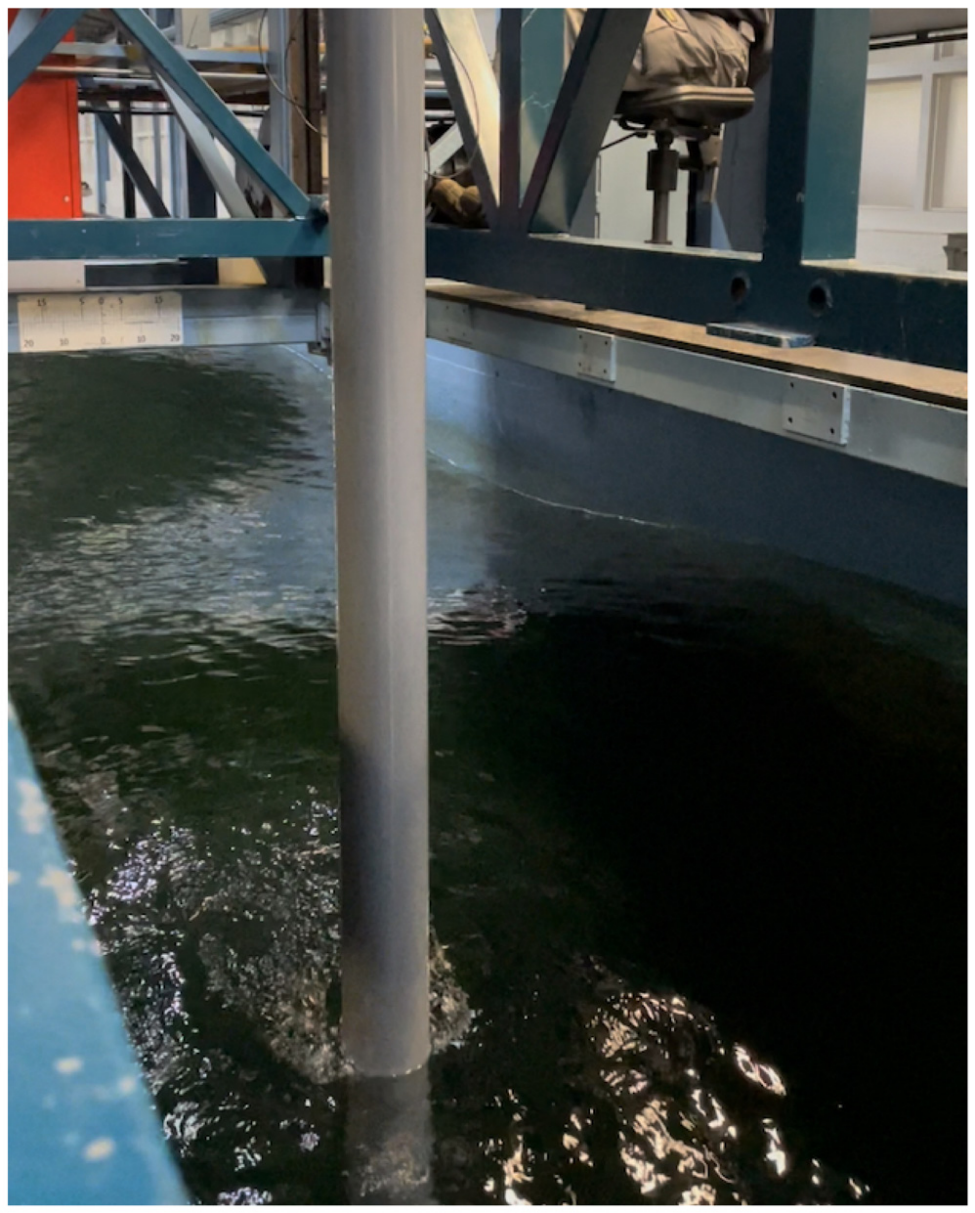
Interactions of regular waves with the beam.

**Figure 12.**
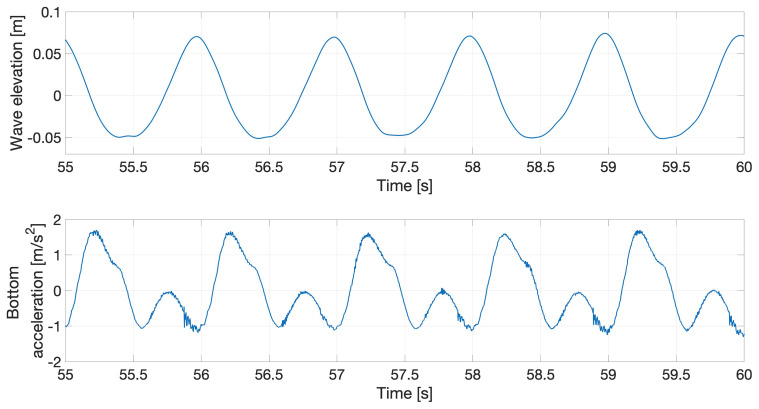
Response of the flexible beam to regular water waves.

Figure 11 depicts the set-up for Case 1, which is further divided into two subcases corresponding to different submerged beam lengths. Subcases 1 and 2 respectively have 0.25m and 0.5m of the beam submerged, and the wave parameters for each subcase are shown in Table 8 and Table 9 respectively, in which *H* denotes the wave height, *T* the wave period and Λ the wavelength; the last column gives the (dimensionless) wave steepness, defined as *H*/Λ. Waves of steepnesses 0.08, 0.04 and 0.03 are generated to interact with the flexible beam. We remark that the wave parameters in Table 8 and Table 9 are those relating to experimental input; parameters gleaned from the actual waves generated in the wavetank were observed to differ from the input ones by up to 5%, as discussed in more detail in Section 8 below. Case 1 aims to validate the linear FSI solvers in the non-resonant regime, as the natural frequencies of the beam are higher than those of the wave. However, some tests with high waves were also performed that excited the beam’s natural frequency due to nonlinear (sum-frequency) effects, as shown in Figure 12, whose two subplots show: (upper) the water wave interacting with the flexible beam; (lower) acceleration, in the *x*-direction, of the submerged end of the beam.

**Table 8.** Input parameters and characteristics of regular waves when the carriage is at rest and 0.25m of the beam is submerged in water.

H	T	Λ	Steepness (H */*Λ)
[m]	[s]	[m]	[-]
0.126	1	1.56	0.081
0.282	1.5	3.51	0.080
0.016	0.5	0.39	0.041
0.062	1	1.56	0.040
0.14	1.5	3.51	0.040
0.25	2	6.239	0.040
0.39	2.5	9.748	0.040
0.016	0.58	0.525	0.031

**Table 9.** Input parameters and characteristics of regular waves when the carriage is at rest and 0.5m of the beam is submerged in water.

H	T	Λ	Steepness (H */*Λ)
[m]	[s]	[m]	[-]
0.032	0.5	0.39	0.082
0.126	1	1.56	0.081
0.282	1.5	3.51	0.080
0.016	0.5	0.39	0.041
0.062	1	1.56	0.040
0.14	1.5	3.51	0.040
0.25	2	6.24	0.040
0.016	0.58	0.52	0.030

## 5. Case-2 experiments: interactions of regular water waves with the flexible beam when the carriage is moving at a constant speed

Case-2 experiments are divided into two subcases, distinguished as in Case 1: wave parameters for the first and second subcases are now shown in Table 10 and Table 11 respectively.

**Table 10.** Input parameters and characteristics of regular waves when the carriage is moving at a constant speed and 0.25m of the beam is submerged in water.

H	T	Λ	Steepness (H */*Λ)	*u*_0_	*ω _e_*
[m]	[s]	[m]	[-]	[m/s]	[rad/s]
0.126	1	1.560	0.081	0.297	7.480
0.016	0.5	0.390	0.041	0.149	14.967
0.062	1	1.560	0.040	0.297	7.480
0.14	1.5	3.509	0.040	0.446	4.987

**Table 11.** Input parameters and characteristics of regular waves when the carriage is moving at a constant speed and 0.5m of the beam is submerged in water.

H	T	Λ	Steepness (H */*Λ)	*u*_0_	*ω _e_*
[m]	[s]	[m]	[-]	[m/s]	[rad/s]
0.126	1	1.56	0.081	-0.215	5.417
0.016	0.5	0.39	0.041	-0.1077	10.831
0.062	1	1.56	0.040	-0.2154	5.415
0.14	1.5	3.51	0.040	0.6864	5.418

**Figure 13.**
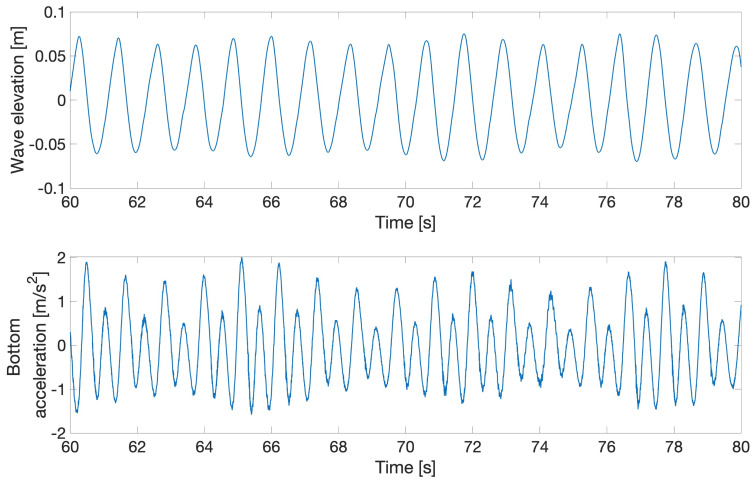
Response of the flexible beam (0.5m submerged) to regular water waves when the carriage is moving at a constant speed.

Moving the carriage changes the frequency with which waves encounter the beam, so that the dynamic response of the beam and its interaction with water waves, particularly at the onset of resonance, can be studied. By changing the steepness of regular waves, both linear and nonlinear FSI solvers can be validated. The encounter frequency *ω _e_* of the waves is calculated as


$$\;\omega_{e}=\omega_{0}\pm u_{0}\frac{\omega_{0}^{2}}{g},\;(12)$$

where *ω*_0_ = 2 *π/T* is the earth-bound frequency of the waves, *u*_0_ is the velocity of the carriage (designated as positive/negative when the carriage moves against/with the waves) and *g* is the gravitational acceleration. Tests are conducted for cases with the carriage moving both with and against the waves. The speed was selected such that the natural frequency was an integer multiple (1,2 or 3) of the encounter frequency. The speed was limited to 0.7m/s because higher speeds introduce loads that would have damaged the experimental set-up. The response of the flexible beam to regular waves with 0.5m of the beam submerged is shown in Figure 13.

## 6. Case-3 experiments: interactions of irregular water waves with the flexible beam when the carriage is at rest

**Figure 14.**
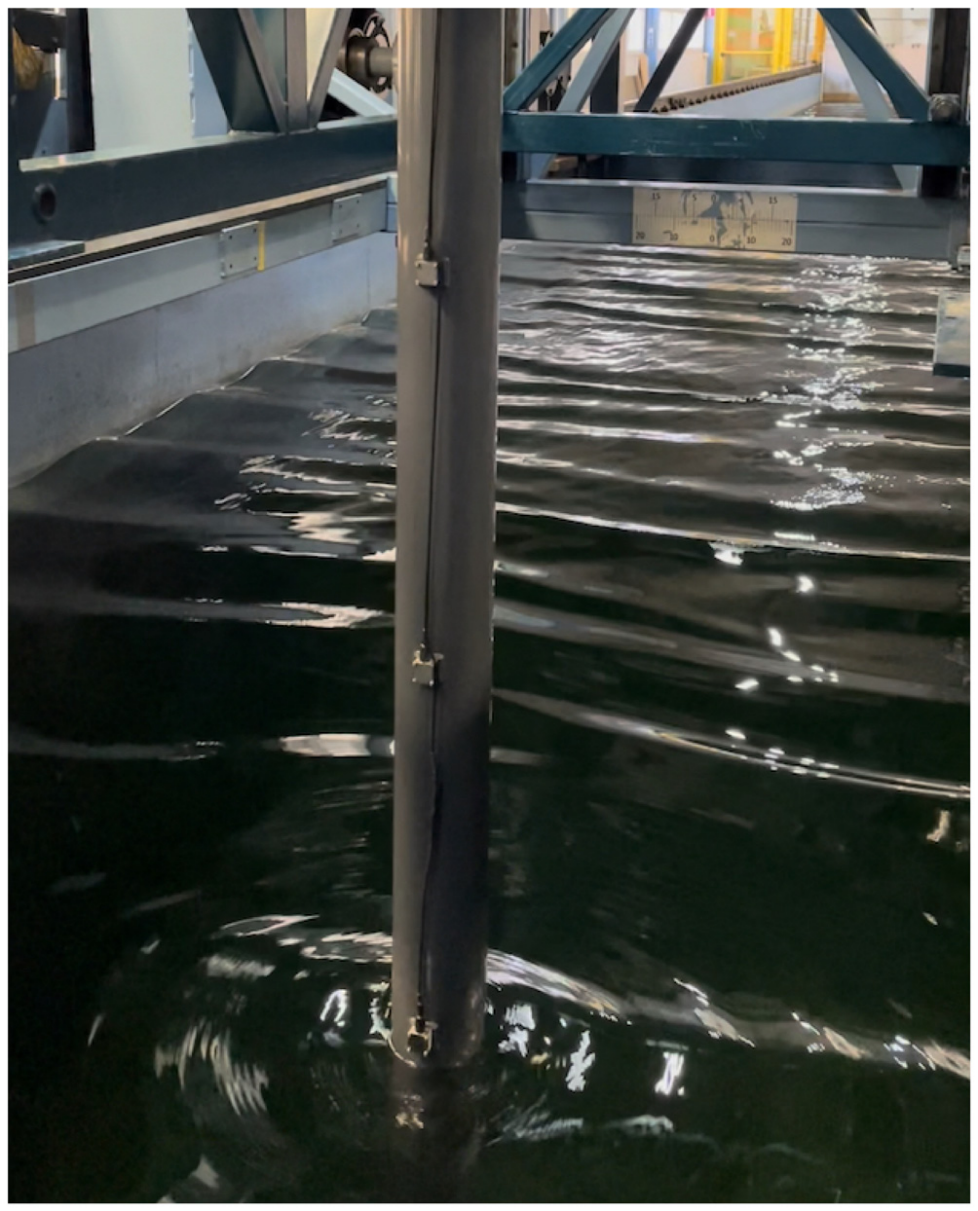
Interactions of irregular waves with the beam.

Case-3 experiments are divided into two subcases, distinguished as in Cases 1 and 2: wave parameters for the first and second subcases are now shown in Table 12 and Table 13 respectively. Case 3, whose experimental set-up is shown in Figure 14, is the most complex of the cases considered and is designed to yield data on structural dynamics due to nonlinear wave-loading processes related to steep and breaking waves. Irregular waves are modelled in the experimental facilities by using already-developed wave-spectrum models, which were developed to replicate oceanographic waves and are given in the form of parameterised functions. There are different models to represent waves in different regions of the world and conditions, i.e. deep seas ^14^, shallow water ^15^, and fully developed seas ^16^. In this study, we have experimentally modelled the JONSWAP (Joint North Sea Wave Observation Project) spectrum ^14^, which represents irregular wave patterns in the North Sea. The parametric equation for the JONSWAP spectrum is given as follows:


$$\;\begin{array}{c} \;S\left(f\right)=\frac{\alpha g^{2}}{{\left(2\pi \right)}^{4}f^{5}}\text{exp}\left(-\frac{5}{4}\left(\frac{f}{f_{m}}\right)\right)\gamma^{\text{exp}\left(\frac{-{\left(f-f_{m}\right)}^{2}}{2\sigma^{2}f_{m}^{2}}\right)},\\ \sigma =\{\begin{array}{c} \sigma_{a}=0.07\;\text{for}\;f\leq f_{m},\\ \sigma_{b}=0.09\;\text{for}\;f>f_{m}, \end{array} \end{array}\;(13)$$

**Table 12.** Input parameters and characteristics of irregular waves when the carriage is at rest and 0.25m of the beam is submerged in water.

MARIN Test No. 70065_02CB_02	Environment	Time	Irregular-Sea Characteristics			
			JONSWAP Type Spectrum			
			H _s_	T _p_	Dir.	*γ*
		[s]	[m]	[s]	[deg]	[-]
**North Sea state**						
011_001_01	Gain 1.0	1781	0.34	2.25	180	2.9
011_001_01	Gain 0.25	1781	0.085	2.25	180	2.9
011_001_01	Gain 0.5	1781	0.17	2.25	180	2.9

**Table 13.** Input parameters and characteristics of irregular waves when the carriage is at rest and 0.5m of the beam is submerged in water.

MARIN Test No. 70065_02CB_02	Environment	Time	Irregular-Sea Characteristics			
			JONSWAP Type Spectrum			
			H _s_	T _p_	Dir.	*γ*
		[s]	[m]	[s]	[deg]	[-]
**North Sea state**						
011_001_01	Gain 1.0	1781	0.34	2.25	180	2.9
011_001_01	Gain 0.5	1781	0.17	2.25	180	2.9

where *f _m_* is the maximum frequency of the spectrum; *g* is gravitational acceleration; *α* is a coefficient, known as the Philips parameter, that scales the overall magnitude of the spectrum and is taken as 0.0081; *γ* is the peak-enhancement factor whose value is region dependent ^17^, e.g. 3.43 to 3.70 for the Jiangsu waters in China ^18^. This case aims to validate high-fidelity FSI solvers.

**Figure 15.**
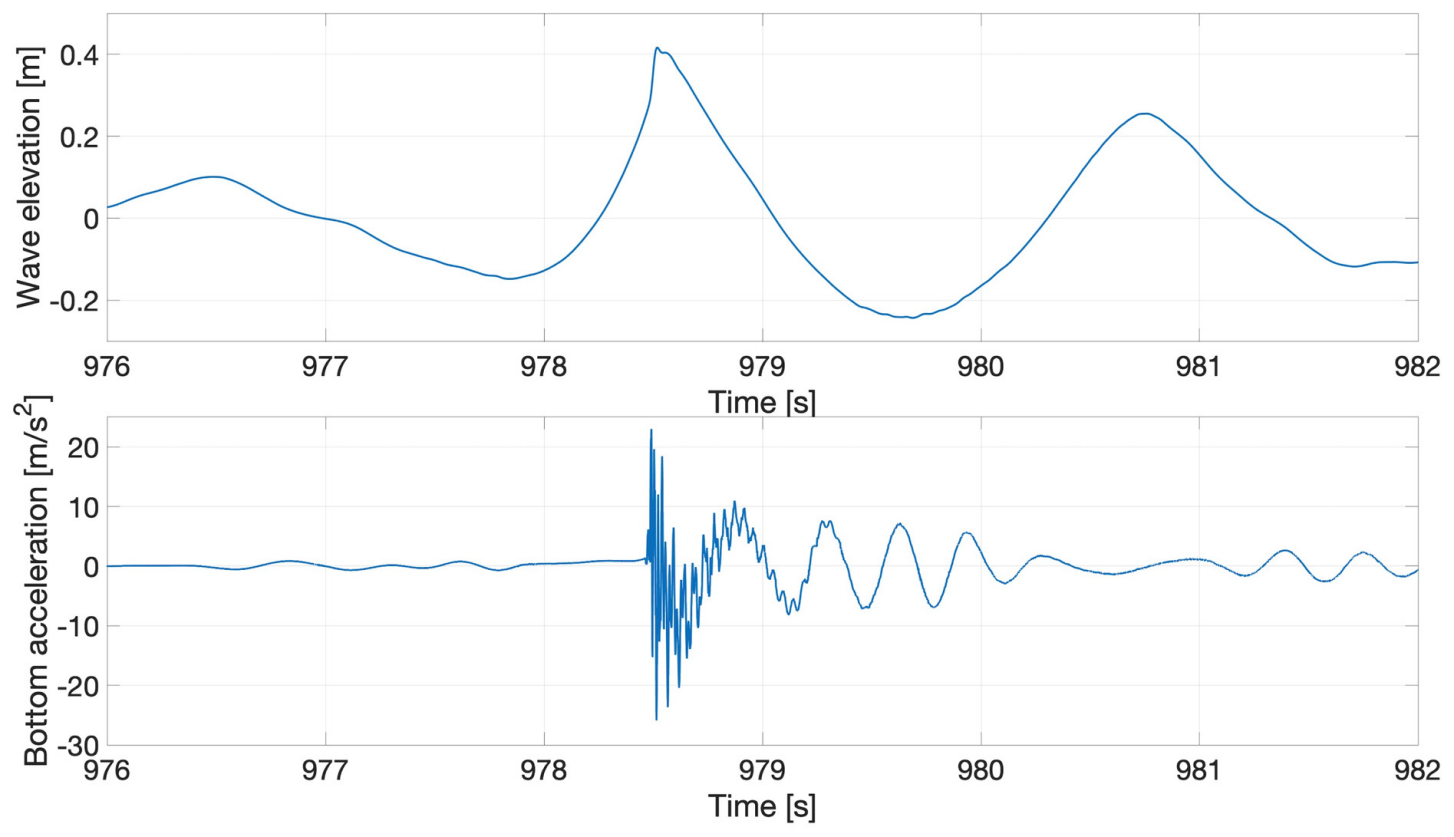
Response of the flexible beam to irregular waves.

**Figure 16.**
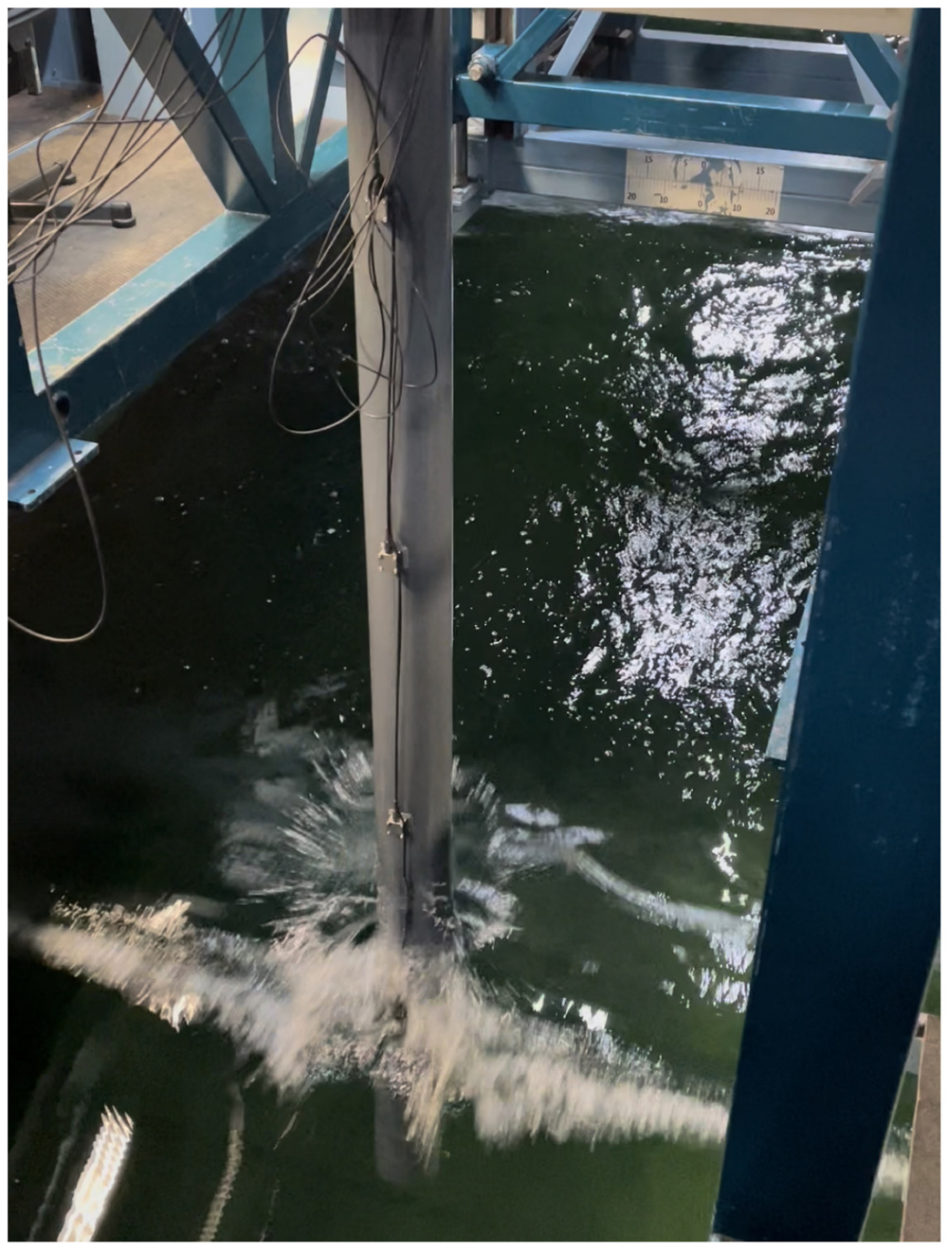
Interactions of irregular waves with the beam.

In Table 12 and Table 13, the Environment parameter Gain 1.0 represents the actual wave spectrum of the North Sea state, whereas Gain 0.25 generates scaled waves up to a quarter of the actual wave height and Gain 0.5 generates waves scaled up to half the actual wave height. *H _s_* is the significant wave height and *T _p_* is the wave period. We report one interesting event that occurred when a steep breaking wave interacted with the beam, whose response is recorded and plotted in terms of the time-varying data shown in Figure 15 and Figure 16.

**Figure 17.**
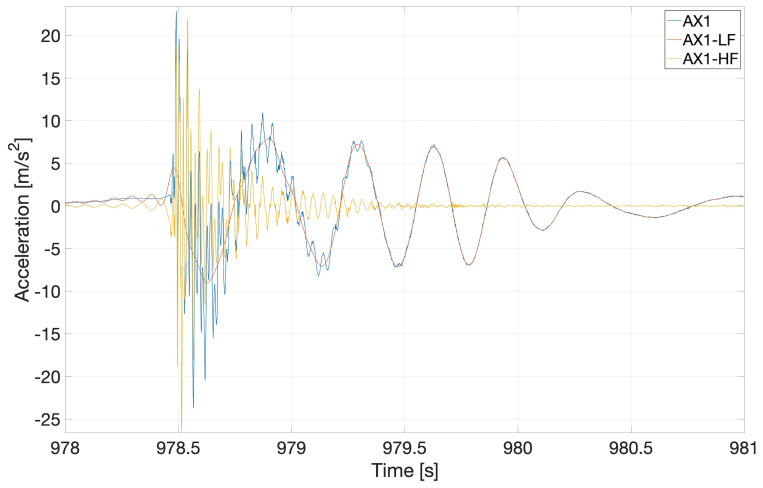
Frequency analysis of the response of the flexible beam to irregular waves. The original signal (blue) is decomposed into higher (yellow) and lower (red) frequency responses. In the legend, AX1 represents the original signal, while AX1-HF and AX1-LF are the respective high-frequency and low-frequency parts of the original signal.

The nonlinear dynamic beam response clearly shows multiple modes, which are further investigated by performing a frequency analysis. The time-domain response is first filtered (using proprietary Matlab software from MARIN) and then decomposed into higher and lower time-domain response-frequency components. The actual and filtered time-domain responses are compared in Figure 17, which reveals that the impact wave excited multiple natural frequencies in the beam. The nonlinear response of the beam is due to the excitation of higher frequencies: in particular, it can be seen that the high-frequency response (yellow) decays faster than the low-frequency response (red) as a result of structural and hydrodynamic damping.

## 7. Availability of data

An open, public-access GitHub and Zenodo ^19^ repositories have been created to share all experimental data ^1^. In addition to the CAD drawing of the beam and clamping, the repository has seven folders, each named as follows (in italics) and containing measurements corresponding to the:

- *hammer_tests* – hammer tests in the form of *.h5 * format files;
- *Exp1_carriage_rest_0.25m* – wave parameters listed in Table 8.
- *Exp1_carriage_rest_0.5m* – measurements corresponding to the wave parameters listed in Table 9;
- *Exp2_carriage_moving_0.25m* – wave parameters listed in Table 10;
- *Exp2_carriage_moving_0.5m * – wave parameters listed in Table 11;
- *Exp3_irreg_waves_0.25m* – wave parameters listed in Table 12;
- *Exp3_irreg_waves_0.5m* – wave parameters listed in Table 13.

All measurements are given in the form of *.h5m* format files, each of which has a corresponding *.pan* format file containing details of measurement names, units, frequency, maximum, minimum and standard deviation. The MATLAB as well as Python scripts for reading the *.h5m* format files and plots presented in the article are also shared.

## 8. Experimental uncertainty

To assess the accuracy and reliability of the experimental data recorded, it is essential to quantify the error at each stage of the experiments. Therefore, this section explains the different types of errors that may affect the measurements, and the precautionary steps taken to minimise them. The experimental campaign can be divided into three stages: designing and fabricating the experimental set-up; performing the experiments and recording the measurements; and, processing the recorded data. Each stage incurs associated specific errors.

In the first stage, errors may accrue through defects in the design and manufacturing. Cooke *et al.* ^20^ presents three case studies that are useful to understand error occurrence during the design stage. Therefore, to minimise this error the set-up was designed and fabricated by the team of researchers and technicians at MARIN and the material was procured from certified providers. The sensors were tested and calibrated, and the set-up was inspected before deploying in the wavetank. During this stage, we found that one of the accelerometers (ACC-2) was defective and was hence replaced by the team. Furthermore, we performed several hammer tests during the experiments to ensure that the structure’s resonance period after the experiments was the same as the initial resonance period before applying the loading. The purpose was to ensure that the beam was not damaged by the water-wave interactions.

The second stage is when actual experiments are performed during the generation of water waves in the wavetank. Before recording actual experimental measurements, the six accelerometers and two probes (see Figure 2 and Section 2) were calibrated. Experimental wave-generation is an iterative process and, from previous experiments, the experts observed that the actual generated-wave parameters in the wavetank can differ by 1% to 5% from the input-wave parameters. These variations occur due to basin effects, i.e., wave reflections. To quantify the discrepancy, we have compared the input-wave parameters and actual waves measured by the probe for the first subcase of experimental case 1. The percentage relative error between the input parameters, i.e. wave amplitude, time period and wavelength and those of the actual wave measured in the basin are listed in Table 14. We note that the percentage relative error ranges from 0 to 6.98. To obtain the values of wave amplitude presented in Table 14, we performed a harmonic analysis of measurements obtained from the probe located in front of the beam. That is, an uninterrupted wave-signal window was selected from the measured wave elevation and then analysed in the frequency domain by performing a Fourier transform. Next, the signal’s amplitude at the corresponding time period was recorded. To minimise this error, we experimentally simulated water waves without the beam set-up and ensured that the generated waves were within the acceptable range i.e. 5% to 6%, in keeping with the above percentage-relative error quantification. Another type of error, arising at the second stage, is the intrinsic instrument error of the measuring equipment. The sensors involved are the two wave probes that measure the incident and reflected waves, and the six accelerometers that measure the beam’s accelerations at six equidistant points. To quantify this type of error, we took measurements twice and then computed the relative difference. Table 15 shows the relative difference in the first fundamental frequency ( *f* ^(1)^) of the beam, measured by the accelerometers, when a dry hammer test was performed twice. The fundamental frequency is computed by taking the Fourier transform of the time-domain signal by using the MATLAB function FFT. Results shown in Table 15 show that the relative difference of the frequencies from the two hammer tests is less than 1%, which confirms that the instrumental errors are dominated by those accruing from wave-generation effects.

**Table 14.** Percentage relative error between the input wave parameters and those of the actual wave generated in the wavetank; here, for the first subcase of experimental case 1.

A	T	Λ
[m]	[s]	[m]
0.00%	-0.50%	-0.99%
2.17%	-0.86%	-1.71%
2.56%	1.01%	2.03%
-5.20%	0.05%	0.10%
1.45%	-0.17%	-0.33%
1.63%	0.03%	0.06%
0.52%	0.40%	0.80%
-6.98%	0.52%	1.04%

**Table 15.** Accelerometer-measurement errors.

	Test 1	Test 2	Relative error%
	*f* ^(1)^ [1/s]	*f* ^(1)^ [1/s]	
AX1	3.6	3.59	0.28%
AX2	3.6	3.59	0.28%
AX3	3.6	3.59	0.28%
AX4	3.58	3.59	-0.28%
AX5	3.56	3.59	-0.84%
AX6	3.6	3.58	0.56%

In the third stage, errors arise in the time-domain processing of experimentally measured data, for example, the signal’s amplitude and frequency. The data-processing error depends upon the algorithm used to analyse the data, e.g. discrete Fourier transform. Other common examples of this type of error are truncation error, overflow error, and rounding error.

In addition to the above-mentioned errors, human error also contributes towards total error; this can be minimised by re-examining both set-up and measurements. Accordingly, we ensured that specialised teams of experts performed relevant parts of the experiments, i.e. design, fabrication, bending test, hammer tests and the actual FSI experiments in the wavetank. Moreover, through our numerical model of the beam (utilising the parameters given in Table 7), we found that the relative error between the experimentally- and numerically-computed dry resonance period is 0.3%. A more detailed comparison of experimental data with numerical results is part of an independent yet related research article.

## 9. Conclusion

This experimental study tested the dynamic response of a flexible beam subjected to a wide range of simulated sea states, namely, mild-to-extreme and regular-to-irregular. The experimental data obtained herein will be useful for mathematical, engineering and computational research communities in the validation of FSI numerical solvers ranging from linear to high-fidelity.

## Data Availability

Source code available from: https://github.com/EAGRE-water-wave-impact-modelling/FSI_Experiments/tree/main Archived source code at time of publication: https://doi.org/10.5281/zenodo.13378870 License: Creative Commons Attribution 4.0 International license (CC-BY 4.0).

## References

[1] Kumar A Weir T : Wind power in fiji: a preliminary analysis of the butoni wind farm.In: *International solar energy society conference*.2008. Reference Source

[2] Ram K Ahmed MR Lee YH : Experimental study of wave forces on an offshore wind turbine tower model.In: *2017 4th Asia-Pacific World Congress on Computer Science and Engineering (APWC on CSE).*IEEE,2017;265–270. 10.1109/APWConCSE.2017.00054

[3] Jonkman J Butterfield S Musial W : Definition of a 5–mw reference wind turbine for offshore system development.Technical report, National Renewable Energy Lab.(NREL), Golden, CO (United States),2009. Reference Source

[4] Salwa TJ : On variational modelling of wave slamming by water waves.PhD thesis, University of Leeds,2018. Reference Source

[5] Salwa T Bokhove O Kelmanson MA : Variational modelling of wave-structure interactions with an offshore wind-turbine mast. *J Eng Math.* 2017;107(1):61–85. 10.1007/s10665-017-9936-4

[6] Yan J Korobenko A Deng X : Computational free-surface fluid–structure interaction with application to floating offshore wind turbines. *Comput Fluid.* 2016;141:155–174. 10.1016/j.compfluid.2016.03.008

[7] Crespo AJC Altomare C Domínguez JM : Towards simulating floating offshore oscillating water column converters with smoothed particle hydrodynamics. *Coast Eng.* 2017;126:11–26. 10.1016/j.coastaleng.2017.05.001

[8] Sassi P Freiría J Mendina M : Simulation of vorticity wind turbines. *Heliyon.* 2020;6(10): e05155. 10.1016/j.heliyon.2020.e05155 33088944 PMC7557875

[9] Jiang Z : Installation of offshore wind turbines: a technical review. *Renew Sust Energ Rev.* 2021;139(44): 110576. 10.1016/j.rser.2020.110576

[10] Bhattacharya S Nikitas G Arany L : Soil-Structure Interactions (SSI) for offshore wind turbines. *IET Eng Technol Ref.* 2017;24(16). 10.1049/etr.2016.0019

[11] MARIN concept basin. Accessed: 19-11-2022. Reference Source

[12] Blevins RD Plunkett R : Formulas for natural frequency and mode shape. *J Appl Mech.* 1980;47(2):461–462. 10.1115/1.3153712

[13] Ewins DJ : Modal testing: theory, practice and application.John Wiley & Sons,2009. Reference Source

[14] Hasselmann K Barnett TP Bouws E : Measurements of wind-wave growth and swell decay during the Joint North Sea Wave Project (JONSWAP). *Ergaenzungsheft zur Deutschen Hydrographischen Zeitschrift, Reihe A.* 1973. Reference Source

[15] Huang NE Long SR Tung CC : A unified two-parameter wave spectral model for a general sea state. *J Fluid Mech.* 1981;112:203–224. 10.1017/S0022112081000360

[16] Pierson WJ Jr Moskowitz L : A proposed spectral form for fully developed wind seas based on the similarity theory of sa kitaigorodskii. *J Geophys Res.* 1964;69(24):5181–5190. 10.1029/JZ069i024p05181

[17] Lee UJ Jeong WM Cho HY : Estimation and analysis of JONSWAP spectrum parameter using observed data around Korean Coast. *J Mar Sci Eng.* 2022;10(5):578. 10.3390/jmse10050578

[18] Feng WB Yang B Cao HJ : Study on wave spectra in south coastal waters of Jiangsu. *Appl Mech Mater.* 2021;212:193–200. 10.4028/www.scientific.net/AMM.212-213.193

[19] Rehman W : Benchmark experimental data: water-wave interactions with a flexible beam [Data set]. Experimental Modeling of Water-Wave Interactions With a Flexible Beam (OMAE2023–108105), Melbourne, Australia. Zenodo.2024. 10.5281/zenodo.13378870

[20] Cooke JA McMahon CA North MR : Sources of error in the design process. *Recent Advances in Integrated Design and Manufacturing in Mechanical Engineering*.2003;421–430. 10.1007/978-94-017-0161-7_41

[21] Rehman W Bunnik T : Fluid-structure interaction modelling of the regular water-waves impact on a flexible beam.In: *Proc ASME Power and Energy Conference.*ASME,2024;10.

[22] Rehman W Bunnik T Bokhove O : Experimental modelling of water-wave interactions with a flexible beam. *EarthArXiv eprints.* 2024; X5998B. 10.31223/X5998B PMC11607480 39618811

