# Supplementary material for: Experimental Modelling of Water-Wave Interactions with a Flexible Beam
Source: Open Res Eur. 2024 Oct 18;4:229. [Version 1] doi: 10.12688/openreseurope.17831.1 (PMC11607480; doi:10.12688/openreseurope.17831.1)
Supplement: Peer review history

# Reviewer report: Reviewer response for version 1

**DOI:** 10.21956/openreseurope.19279.r45872

**Published:** 2024-11-29

**Authors:** Zhaolong Yu

**Affiliations:** 1 Norwegian University of Science and Technology, Trondheim, Norway

The paper presented useful experimental results of wave interactions with a partly submerged free-clamped beam that can be useful for understanding the problem and potential benchmark studies in the future. A few suggestions and questions arise.

 1. The experiments were carried out with the free end approaching the water. This seems to be upside down compared to a fixed OWT. what is the consideration here?

 2. It is not clear how the beam dimensions are determined. Is there a specific turbine the authors are trying scaling down for the tests?

 3. The 0.25m wet hammer test curve in Fig. 6 seems very different from the other two. Why?

 4. All the curves in Fig. 6 should be zoomed in to be meaningful.

 4. The beam modes in Fig 8 are suggested to be normalized for better illustration.

 5.  The mass of RNA is important for a bottom fixed OWT with respect to the mode shapes and natural frequencies, but was not considered here. Any comments on this?

Is the study design appropriate and does the work have academic merit?

Yes

Is the work clearly and accurately presented and does it cite the current literature?

Yes

If applicable, is the statistical analysis and its interpretation appropriate?

Yes

Are all the source data underlying the results available to ensure full reproducibility?

Yes

Are the conclusions drawn adequately supported by the results?

Yes

Are sufficient details of methods and analysis provided to allow replication by others?

Yes

Reviewer Expertise:

marine structures

I confirm that I have read this submission and believe that I have an appropriate level of expertise to confirm that it is of an acceptable scientific standard.

# Reviewer report: Reviewer response for version 1

**DOI:** 10.21956/openreseurope.19279.r45477

**Published:** 2024-11-06

**Authors:** Changqing Jiang

**Affiliations:** 1 University of Duiburg-Essen, Duisburg, Germany

This manuscript offers a valuable and comprehensive contribution to the field of wave-structure interactions (WSI), focusing on experimental measurements of wave interactions with a flexible beam under varied sea conditions. Through systematic capture of beam deflections and wave interactions across a wide range of conditions, from regular to irregular and from moderate to extreme, the authors have produced a robust dataset that facilitates the validation of fluid-structure interaction (FSI) solvers, spanning from linear to high-fidelity models.

 However, I have a few questions for clarification. First, were the accelerometers used in this experiment single-axis or multi-axis? Additionally, could the authors clarify if these accelerometers are intended to measure proper acceleration or coordinate acceleration, as Figure 2 describes two coordinate systems?

 Finally, I am curious about the discrepancy in the wet hammer test results at 0.25 m, which appear significantly different from the other two measurements. Given that 0.25 m is the midpoint between 0 m and 0.5 m, one would expect it to yield results more similar to those two.

 Thank you for openly sharing this valuable dataset.

Is the study design appropriate and does the work have academic merit?

Yes

Is the work clearly and accurately presented and does it cite the current literature?

Yes

If applicable, is the statistical analysis and its interpretation appropriate?

I cannot comment. A qualified statistician is required.

Are all the source data underlying the results available to ensure full reproducibility?

Yes

Are the conclusions drawn adequately supported by the results?

Yes

Are sufficient details of methods and analysis provided to allow replication by others?

Yes

Reviewer Expertise:

wave-structure interactions

I confirm that I have read this submission and believe that I have an appropriate level of expertise to confirm that it is of an acceptable scientific standard.
